# Soft Tissue Augmentation Techniques in Implants Placed and Provisionalized Immediately: A Systematic Review

**DOI:** 10.1155/2016/7374129

**Published:** 2016-07-19

**Authors:** Rosa Rojo, Juan Carlos Prados-Frutos, Ángel Manchón, Jesús Rodríguez-Molinero, Gilberto Sammartino, José Luis Calvo Guirado, Rafael Gómez-de Diego

**Affiliations:** ^1^Department of Stomatology, Faculty of Health Sciences, Rey Juan Carlos University (URJC), Avenida Atenas s/n, 28922 Madrid, Spain; ^2^Department of Stomatology and Maxillofacial Surgery, Federico II University, Corso Umberto I 40, 80138 Naples, Italy; ^3^Department of International Dentistry Research, Faculty of Medicine and Dentistry, San Antonio Catholic University of Murcia (UCAM), Avenida Jerónimos 135, 30107 Murcia, Spain

## Abstract

The aim of this study was to evaluate the effectiveness of techniques for soft tissue augmentation in the placement of immediate implants with and without provisionalization and to assess the quality of the reports in the literature. Randomized clinical trials, prospective clinical trials, and case series were included in this review. Clinical questions were formulated and organised according to the PICOS strategy. An electronic search was performed in PubMed, Cochrane Central Register of Controlled Trials, Scopus, and ISI Web up until June 2016. Interexaminer agreement on eligibility (*k* = 0.842; *p* = 0.103) and quality (*k* = 0.933; *p* < 0.001) was high. Methodological approaches were assessed using criteria based on design related forms designed by the Dutch Cochrane Collaboration. Finally, 14 papers were identified. In two studies, the implant survival was 90%; for the rest of the studies it was 100%. All studies reported favourable aesthetic, biological, and radiographic outcomes. Surgical and biomechanical complications of this technique were not relevant. This technique effectively compensates for the expected loss of volume of the oral soft tissues and maintains high success rates with good aesthetic results over time.

## 1. Introduction

After tooth extraction, a number of changes take place in the socket during the following 12 months of surgery. The width of the ridge will be reduced by 50% (about 5 to 7 mm); two-thirds of this reabsorption occurs after the first three months [1]. These changes expressed both horizontally and vertically are expected in hard and soft tissues [2]. However, further bone loss occurs horizontally and increased resorption of the vestibular cortical thickness [3] results in a more palatal position after the alveolar ridge resorption process [4].

Immediate implant placement (IIP) reduces alveolar resorption [5, 6], the number of surgeries, and the waiting time necessary until the placement of the final restoration [7, 8]. Surgeons should however consider many factors to achieve success in the treatment such as the location of the implant, gingival marginal position, width and thickness of keratinized mucosa [9], gingival biotype [10], vestibular cortical thickness, and the size of the horizontal “gap” buccal or sagittal position of the root [11].

To optimally preserve tissue, surgeons implement IIP [12] to maintain bone architecture and immediate provisionalization to maintain soft tissue [13]. However, the appearance of gingival recession has been reported after the first year in the vestibular cortex [14–16]. To increase thickness of the gingival tissues practitioners have suggested using connective tissue grafts (ITC) as a method of preservation of soft tissue levels [12, 17, 18].

A connective tissue graft associated with IIP was described by Edel [19] who used a biological membrane to cover the residual alveolar defects associated with tooth extraction and considered it a valid protocol [20, 21] against the use of synthetic membranes that show more clinical complications, such as colonization and bacterial infection [22, 23]. Biological membranes also improve metabolic environment of the local soft tissue surface preserving the amount of keratinized tissue and allow for optimal marginal and peri-implants seals [24]. We have therefore developed an associated surgical procedure based on IIP palatal flaps rotation to obtain and maintain coverage of primary soft tissue and crestal bone augmentation following placement of the IIP [25, 26].

Some systematic reviews report the effectiveness of soft tissue augmentation procedures around dental implants and in partially edentulous sites [27] and changes in mucosal soft tissue thickness and keratinized mucosa width after soft tissue grafting around dental implants [28] or evaluate success, the function, complications, and patient satisfaction between “immediate,” “immediate-delayed,” and “delayed” implants [29]. However, there is still a limited number of articles analysing this type of mucogingival technique in the immediate implants. The aims of this review were (1) to evaluate the effectiveness of mucogingival techniques after extraction and implant placement in adult patients in randomized controlled trials (RCT), prospective clinical trials (PCT), and case series (CS) and (2) to analyse the changes in aesthetic and clinical outcomes in the peri-implant tissues.

## 2. Material and Methods

The reporting of this systematic review is based on the PRISMA guidelines [30]. A structured approach was used to formulate the research question for this systematic review using five components commonly known by the acronym “PICOS” [31]: the patient population (P), the interventions (I), the comparison group (C), the outcome of interest (O), and the study design (S).

We therefore chose studies that demonstrated this:

 Participants: adult patients who needed a dental extraction and who required an immediate implant treatment.
 Interventions: use of connective tissue graft alone or combined with immediate provisionalization and use of rotated palatal graft.
 Comparisons: no use of mucogingival and immediate restoration techniques.
 Outcome: changes in aesthetic and clinical outcomes.
 Study design: randomized controlled trials (RCT), prospective clinical trials (PCT), or case series (CS).

### 2.1. Search Strategy

**Figure 1:**
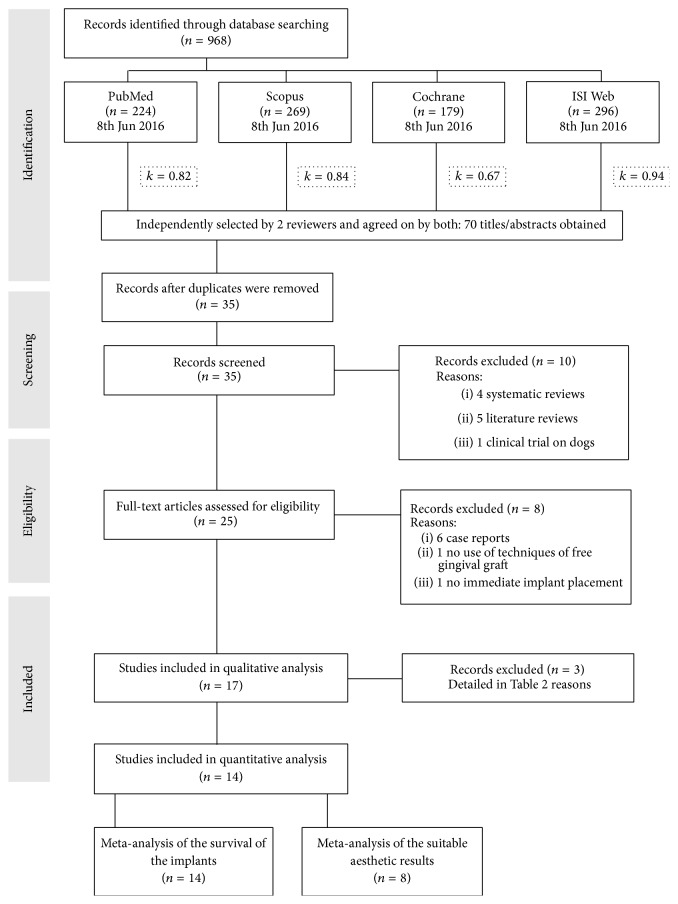
Flow chart of the literature search.

An electronic search was performed in PubMed, Scopus, ISI Web, and the Cochrane Oral Health Group Specialized Trials Register (RCTs) database until the 21st of May 2015. Two authors (Rosa Rojo and Jesús Rodríguez-Molinero) performed all searches and selected articles fulfilling the inclusion criteria independently and in duplicate (Figure 1). The level of agreement between the reviewers regarding study inclusion was calculated using Cohen's kappa statistic.

### 2.2. Search Terms

The following search algorithm was used: ((dental (MeSH) OR immediate (MeSH)) AND implants (MeSH)) AND (“connective tissue graft” (free text word) OR “free gingival graft” (free text word) OR “rotated palatal flap” (free text word)) OR “immediate provisionalization” (free text word) OR “immediate implant restoration” (free text word).

### 2.3. Inclusion and Exclusion Criteria

The included study articles had to fulfil all the following criteria: (1) randomized and controlled clinical trials, prospective clinical trials, or case series; (2) at least ten human participants; (3) full-text no language restrictions; (4) studies that carried out immediate placement of dental implant(s); (5) use of connective tissue and/or pedicle flap techniques; (6) with or without immediate provisionalization; (7) the use of any type of graft material.

Reviews and case reports were excluded from this study.

### 2.4. Assessments of Study Quality

Following the selection of eligible papers on the basis of inclusion and exclusion criteria, studies were rated on their quality. Specific study design related forms were designed by the Dutch Cochrane Collaboration based on the Cochrane Handbook for Systematic Reviews of Interventions [41]. We developed a checklist for each study type, focusing on randomization (if applicable), patient and site characteristics, patient selection, intervention, evaluation method, outcome, and follow-up (Table 1).

Two investigators (Rosa Rojo and Jesús Rodríguez-Molinero) independently generated a score for all selected articles expressed as the amount of plus signs given. A score of at least 8 was considered methodologically acceptable for RCTs and that of 7 was acceptable for PCT and CS. To reduce the risk for bias as much as possible, studies showing poor quality on the basis of this assessment were excluded.

### 2.5. Statistical Analysis

A Cohen's kappa statistic was used to evaluate interexaminer agreement on study eligibility and quality.

Due to the heterogeneity between the techniques used in the studies (flapless/graft), a meta-analysis on the survival rates of implants and the rate of suitable aesthetic levels was performed.

Survival rates were calculated by dividing the number of events (survival of the implants or suitable aesthetic results) in the numerator by the total exposure time obtained in the denominator, which is calculated by taking the sum of exposure time of implants that survived the total follow-up time, exposure time up to the failure of implants lost during the observation time, and exposure time up to the end of follow-up time for implants that did not complete the observation period due to any reason.

To evaluate the suitable aesthetic results only studies that had applied aesthetic indexes are included.

The total number of survival rates was considered to be Poisson distributed and Poisson's regression with a logarithmic link function was used. Standard errors were calculated to obtain 95% confidence intervals (CIs) of the summary estimates of the survival rates.

To assess heterogeneity of the study-specific event rates, *I* ^2^ statistics was done and also the *p* value was calculated. If *p* < 0.05, indicating heterogeneity, random-effects Poisson's regression was used to obtain a summary estimate of the survival rates. Survival proportions were calculated by the relationship between survival rate and number of implants evaluated.

All analysis were done using R version 3.1.3 (R Core Development Team, R Foundation, Vienna, Austria) with the interrater reliability (irr) package and metafor package.

## 3. Results

### 3.1. Search Results

All search strategies yielded 738 papers. Two investigators (Jesús Rodríguez-Molinero and Rosa Rojo) independently identified 17 potentially eligible papers. Interexaminer agreement on study eligibility was high (*k* = 0.842, *p* = 0.103). Eligible studies were methodologically assessed by the same investigators with high agreement (*k* = 0.933, *p* < 0.001). Three studies [7, 32, 33] did not meet the inclusion criteria and were excluded. The reasons for exclusion are depicted in Table 2.

One examiner (Rosa Rojo) extracted all data from the selected papers. Finally, 14 papers could be identified. The characteristics of included studies are described in Tables 3 and 4. Nine studies were case series, two were prospective clinical trials, and three were randomized clinical trials. Clinical requirements to be met by the patient are detailed in Table 5.

### 3.2. Study Investigations

Only two of the studies used the rotated palatal flap (RPF) as a technique for increasing the soft tissue and the rest used subepithelial connective tissue graft (SCTG). One study evaluated the long-term effectiveness over a period of up to nine years and one showed clinical efficacy of implant placement for the treatment of nonsalvageable teeth that showed gingival recession or absence of attached gingiva. The parameters evaluated in the studies varied; examinations included the changes in the soft and hard tissues [17, 18, 21, 34, 36–38] and in the all tissue response to the peri-implant [39, 40]. Aesthetic results [35] and rates of success [18, 21, 37–40] also differed between studies.

Also, Nemcovsky et al. [25] reported the use of palatal coverage rotational flap without using membranes to regenerate the crestal bone and which surgical approach was used to allow primary closure [26].

### 3.3. Preoperative and Postoperative Care

*Preoperative*. Some studies reported various preoperative treatments, such as oral administration of an antibiotic one hour prior to surgery, for example, 2 g amoxicillin or 600 g clindamycin for patients allergic to penicillin [17] or 500 mg amoxicillin four times daily for 4 days [20].

*Postoperative*. Patients were instructed to rinse twice daily with chlorhexidine digluconate [17] (0.12% [12, 18, 26, 39, 40] or 0.2% [35]) and to refrain from removing plaque by mechanical means at the surgical site for 2 weeks [39, 40]. Sutures were removed 2 weeks postoperatively, and patients were asked to commence plaque removal at the provisional crown with a soft-bristled toothbrush [17].

Antibiotics and an analgesic were prescribed [18, 35, 38] such as system antibiotics (amoxicillin 625 mg + clavulanic 125 mg two times daily [36] or amoxicillin 500 mg thrice daily for 5 days [21]) and nonsteroidal anti-inflammatory medication (aceclofenac 100 mg two times daily [36] or diclofenac sodium + serratiopeptidase combination thrice daily for 3 days [21]).

A liquid diet was suggested for 1 or 2 weeks [18, 38–40] following surgery with a transition to a soft diet for the next 3 months [18, 38–40].

### 3.4. Implant Survival

In two studies, the implant survival rate was 90% [39, 40]. One implant developed a periapical infection 3 weeks after implant placement [39] and the other patient experienced an early implant failure at the 3-month follow-up appointment due to mobility [40]. The implant survival in the rest of the studies was 100%.

**Figure 2:**
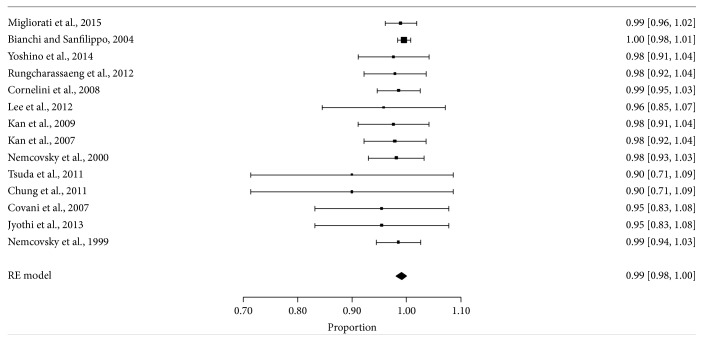
Forest plot for survival rate of proportion of implants evaluated. Proportion ratio corresponds to the percentage of survival implants between the total number of implants evaluated.

In meta-analysis, the annual survival rate of the implant was estimated at 6,526 (6,125–6,927) per 100 years for model of random effects (*I* ^2^ = 93,21%) (Figure 2) translating into the survival of implant as observed in Table 6.

The meta-analysis shown in the forest plot (Figure 2) shows survival proportion of the number of implants evaluated.

### 3.5. Aesthetic Outcomes

The evaluation of the aesthetic results was assessed using the stability of the keratinized mucosa width (KMW) parameters. The mean values of KMW >3 mm were considered acceptable for aesthetic purpose. All patients treated by immediate implant combined with subepithelial connective tissue graft had a KMW value >3 mm at the end of each of the studies' follow-up periods [20, 21, 36]. Stability of this tissue during the 9-year period was reported in one publication [6].

In the experimental groups of studies, none reported aesthetic compromises and, overall, the aesthetic outcomes were quite favourable [7]. In some studies, pink aesthetic score (PES) and white aesthetic score (WES) indexes were used by two independent evaluators. The first evaluates the mesial papilla, distal papilla, curvature of the facial mucosa, level of the facial mucosa, and root convexity/soft tissue color and texture at the facial aspect of the facial implant site as five variables. The second index evaluates the visible part of the implant restoration such as general tooth form, outline and volume of the clinical crown, color, surface texture, translucency, and characterization. In both, a score of 2, 1, or 0 is assigned to each parameter. Thus, in case of an implant restoration, a maximum total PES or WES of 10 is possible. We derived a mean pink aesthetic score (PES) of 7.15 (SD: 1.75) and a mean white aesthetic score (WES) of 7.98 (SD: 0.99). A statically significant difference between control and test groups was revealed for PES scores (*p* < 0.001) while no differences were revealed for WES (*p* = 0.88) [17].

For aesthetic reasons, 1 mm was the maximum discrepancy accepted for attesting to a good alignment of emergence lines (ELs) of the prosthetic crown. The collected data demonstrated a complete success in the 1–3-year test group, while a mean of 80% of the control group showed scores of EL > 1 mm. A low decrement of mean EL scores > 1 mm was reported in the following 6-year interval in both groups [6].

### 3.6. Biological Parameters

The modified plaque index (mPI) demonstrated scores of 0 and 1. There was no statistically significant difference in the mPI at the end of the follow-up period (*p* > 0.05) [39, 40].

All sites that showed a probing depth (PD) value < 3 mm were considered healthy. Covani et al. [20] results showed a mean decrease of PD value between the baseline measurements and the PD value at the end of the follow-up period [21].

After 12 months of surgery, more than 50% of the papilla fill was observed in 80% [18, 39] and 89% [40] of all sites. The papilla index score (PIS) ranged from 0 to 3 at all the time intervals in the studies of Yoshino et al. [18] and Chung et al. [40]. In other studies, the PIS ranged from 2 to 3 [37, 39]; for Lee et al. [36], the PIS ranged from 1 to 3 at all time intervals. No statistically significant differences were noted for either mesial or distal papilla levels among the time intervals and between the test and control groups (*p* > 0.05).

The intraclass correlation coefficient (ICC) for facial gingival level (FGL) measurements was 0.92 [39] and 0.998 [40], indicating that the measurement method was reliable and reproducible. The mean FGL change at the end of the follow-up period was −0.05 mm [39, 40], +0.13 ± 0.61 [37], and −0.25 ± 0.35 [18] (*p* > 0.05). No statistically significant differences for FGL were noted between any of the time intervals.

The mean of periotest values (PTV) at T3 (−2.6 ±  −5.5) [39] (−2.0 ± 0.9) [40] was statistically significantly lower than that at T1 (−0.2 ± 3.8) [39] (−0.1 ± 2.2) [40] (*p* < 0.05), which indicated good stability for the implants.

### 3.7. Radiographic Results

There were no significant differences in marginal bone level (MBL) or in the MBL change either at or between any time intervals between the test and control groups (*p* > 0.05) [18].

The intraclass correlation coefficient (ICC) for MBL measurements was 0.99 [39] and 0.955 [40], indicating that the measurement method was reliable and reproducible. The mean value of the MBL was 1.5 ± 0.5 mm [20], 0.1 ± 0.6 mm [17], +0.10 mm [39], and −0.31 mm [40] at the end of the follow-up period.

### 3.8. Complications

All studies showed generally intraoperative and postoperative complications. In the surgical phase, they reported complications of rotational instability observed in any implants [18, 39] and partial necrosis of the SCTG [39, 40]. Eventually, immediate postsurgical bleeding in the palate occurred and there were granules of the grafting material exfoliating at this position during the first healing weeks [26].

In the prosthetic phase, studies reported the following complications: episodes of provisional restoration debonding [18, 38, 40], fractures in the provisional restorations [40], abutment screw loosening [39, 40], and a fistula tract as a result of the residual flow of composite resin [18, 39].

### 3.9. Survival Suitable Aesthetic Results

In the meta-analysis only those studies whose indexes include applied information were available in a follow-up of 12 months.

PIS indexes of 5 studies [35–37, 39, 40] were included, considering those unfavorable aesthetic results scores equal to or less than 2; PES index of Migliorati et al. [17] was included, considering those unfavorable aesthetic results scores equal to or below 5; the FGL of 2 studies [18, 38] was included, considering unfavorable aesthetic results whose measurements were greater than 1.5 mm.

The annual aesthetic suitable rate of the implant was estimated at 1,292 (1,029–1,555) per 5 years for model of random effects (*I* ^2^ = 64,32%) translating into the survival of implant as observed in Table 6.

**Figure 3:**
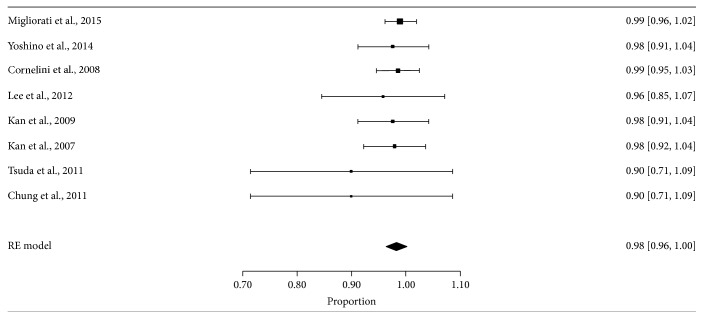
Forest plot for proportion of suitable aesthetic results rate of implants evaluated. Proportion ratio corresponds to the percentage of suitable aesthetic results between the total number of implants evaluated.

The meta-analysis shown in the forest plot (Figure 3) of the proportion of suitable aesthetic results of the number of implants evaluated.

## 4. Discussion

In this paper, we have presented a systematic review of studies that demonstrate techniques for implant placement after tooth extraction. We examined those studies that use autologous connective tissue graft or a rotational palatal flap as options for effective treatment that would compensate for the expected loss of volume labial soft tissue and maintain good aesthetic results over time.

The results showed that all of the studies reported positive behaviour of soft tissue and bone peri-implants. This technique could minimize facial gingival recession; accordingly, several studies observed an increase in gingival tissue of 0.07 mm [37], 0.2 mm [35], 0.25 mm [39], 0.4 mm [36], or 0.5 mm [38]. The use of connective tissue grafts seems to prevent induced complications by using synthetic membranes, improving metabolism in the local environment of superficial tissues [21], and by increasing the height and thickness of the tissue [21, 36, 38], especially if the implants are positioned palatally [20, 34, 38, 39]. This is especially useful in cases of insufficient soft tissue and transformation of a thin gingival biotype to a thick one [37], allowing a sufficient thickness of peri-implant to hide various underlying restoration materials (titanium, titanium-ceramic, zirconia ceramic, and zirconia) [34].

Performing the technique with a rotational palatal flap showed predictable results with or without the use of membranes and was advantageous because it retains some of the blood supply [25]. However, this procedure is not advisable when the probing of the palatal gingiva measured <4 mm [26].

Several factors associated with bone resorption have been reported as due to flap elevation; although some studies [17, 42] argue that lack of flap elevation does not prevent reabsorption, it is vitally important to choose a suitable profile of the patient. The thickness of the bones can also determine the degree to which vertical resorption is produced [38, 40, 43]. Sites with thinner facial bone underwent significantly more vertical resorption than sites with thicker facial bone. The major benefit of this treatment is the preservation of the existing papillae with no risk of creating scar tissue.

The studies reported good results in the absence of using provisional restorations [37]. However, in the study of Yoshino et al. [18], the experimental group receiving the subepithelial connective tissue graft and provisionalization experienced fewer changes in facial gingival levels compared to those not receiving the connective tissue graft.

Although the influence of oral hygiene on implant success has been controversial, it is generally agreed on that plaque accumulation could induce a negative response in the mucosa without a good level of oral hygiene [37, 40]. To minimize disruption to the peri-implant gingival tissue and ISTC teeth immediately after replacement, patients were advised to thoroughly rinse with chlorhexidine solution but refrain from brushing the surgical site for one month following the procedure [39].

In this systematic review, eligible studies were rated on their quality using specific study design related forms designed by the Dutch Cochrane Collaboration. This method was also used by Den Hartog et al. [44] to evaluate the outcome of immediate, early, and conventional single implant treatment. Note that other checklists based on the CONSORT statement for RCTs [45] or STROBE statement for case series [46] could also have been used to evaluate methodological background. Albeit one search method may be considered more detailed than another, we believe that the two papers we excluded would have been omitted in any quality assessment as clear data on the outcome were missing.

Randomized clinical trial, prospective clinical trials, and case series studies were included in this meta-analysis to summarize data on survival rates and the failure of the implants with these techniques.

After the period of investigation, the highest rates of failure (10%) were observed in studies of Tsuda et al. [39] and Chung et al. [40]. However, the sample size was insufficient (10 implants per study) and due to heterogeneity between variables (flap technique, graft, provisionalization, and using membrane) it was not possible to determine whether any of them could affect the survival of the implant.

For the meta-analytic study of soft tissues higher rates of failure (70%) were observed in the study of Tsuda et al. [39], where the implant failure is also accompanied, which could be due to an inadequate assessment of the clinical variables intervention protocol, since due to heterogeneity between variables it was not possible to determine if any of them could have significantly affected the results.

Studies Kan et al. [38] and Cornelini et al. [35] are the only ones that combine the technique with flap and flapless, with unfavorable cosmetic results (68% and 65%, resp.). However, studies of Yoshino et al. [18] and Lee et al. [36] (with failure rates of 0% and 30%, resp.) do not employ flap, but the study of Kan et al. [37] (with aesthetic failure rate of 16%) is used. In all grafts, they are used. These results suggest that the use of the flap does not seem to influence the final results.

Immediately placing the implant is especially the most advanced treatment modality, reducing the number of surgical treatments and the time between tooth extraction and positioning of the definitive prosthesis. The option presented by this systematic review is an effective means to compensate for the expected loss of lip volume of soft tissue that maintains good aesthetic results over time. It is a simple, safe, and reliable method to achieve functional and aesthetic restorations with a high degree of success [6, 17, 20, 34, 35].

However, stabilization of tissues is documented in the last period of 6–9 years, so most retrospective studies would be desirable to support the predictability of positive change at the level of the soft and hard tissues.

## Figures and Tables

**Table 1:** Checklist for quality assessment. N/A: not applicable; *∗*: items applicable to prospective controlled clinical trial.

Quality assessment of randomized controlled trials (^*∗*^prospective clinical trial)	Quality assessment of case series
*Randomization*	N/A
(1) Were adequate methods used for randomization? (^*∗*^N/A)	

*Patient and site characteristics*	*Patient and site characteristics*
(1) Were patient characteristics well described for both groups?^*∗*^	(1) Were patient characteristics well described?
(2) Were site characteristics well described for both groups?^*∗*^	(2) Were site characteristics well described?
(3) Were there no disparities in terms of patient or site characteristics between the groups?^*∗*^	

*Patient selection*	*Patient selection*
(1) Were the inclusion and exclusion criteria well described and the same for both groups?^*∗*^	(1) Were the inclusion and exclusion criteria well described?
(2) Did the study report consecutively treated patients?^*∗*^	(2) Did the study report on consecutively treated patients?

*Intervention*	*Intervention*
(1) Were interventions for both groups clearly described?^*∗*^	(1) Was the intervention clearly described?
(2) Were all patients of the same group treated according to the same intervention?^*∗*^	(2) Were all patients treated according to the same intervention?

*Evaluation method*	*Evaluation method*
(1) Was blinding used to assess the outcome?^*∗*^	(1) Was the outcome assessed by an investigator who had not been involved in the treatment?
(2) Were adequate methods used to assess the outcome?^*∗*^	(2) Were adequate methods used to assess the outcome?
(3) Were reproducibility data reported on the outcome variable(s)?^*∗*^	(3) Were reproducibility data reported on the outcome variable(s)?

*Outcome & follow-up*	*Outcome & follow-up*
(1) Was the outcome clearly described?^*∗*^	(1) Was the outcome clearly described?
(2) Was an intention-to-treat analysis performed and was there low risk for selective loss to follow-up?^*∗*^	(2) Was the response rate acceptable and was the number of patients lost to follow-up clearly described?

**Table 2:** Studies excluded after quality assessment and reasons for exclusion.

Authors	Study design	Reasons for exclusion
Grunder et al. [7]	Case series	The inclusion and exclusion criteria were not clearly described; it is unclear whether patients were consecutively treated; outcome was possibly assessed by an investigator involved in the treatment; methods used to assess the outcome were unclear; no actual data on the outcome were available.

Fagan et al. [32]	Case series	Patients characteristics were incomplete (teeth not described); site characteristics were incomplete (age not described); patients were not treated according to same intervention (delayed or immediate implant placement); outcome was possibly assessed by an investigator involved in the treatment; methods used to assess the outcome were unclear; no reproducibility data were reported; no actual data on the outcome were available.

Reinhardt [33]	Case series	Patients characteristics were incomplete (conditions around teeth not described); site characteristics were incomplete (age not described); it is unclear whether patients were consecutively treated; outcome was possibly assessed by an investigator involved in the treatment; no actual data on the outcome were available.

**Table 3:** Characteristics of included studies. RCT: randomized clinical trial; PCT: prospective clinical trial; CS: case series; f: female; m: male; mo: months.

Authors, year	Study design	Follow-up (mo)	Country	Teeth	Sample	Mean age (range of years)	Number of implants evaluated
Migliorati et al., 2015 [17]	RCT	24	Genova, Italy	14 to 24	48 (25 f/23 m)	47,5 (22 to 70)	47
Bianchi and Sanfilippo, 2004 [6]	RCT	72 to 216	Milano, Italy	17 to 27 and 37 to 47	116 (58 f/58 m)	45,4 (19 to 73)	116
Yoshino et al., 2014 [18]	RCT	12	Loma Linda, CA	14 to 24	20 (13 f/7 m)	52,6 (27 to 87)	20
Rungcharassaeng et al., 2012 [34]	PCT	17	Loma Linda, CA	13 to 23	24 (11 f/13 m)	45,4 (23 to 87)	23
Cornelini et al., 2008 [35]	PCT	12	Rimini, Italy	15 to 25 and 35 to 45	34 (15 f/19 m)	43 (21 to 62)	34
Lee et al., 2012 [36]	CS	24	Seoul, Korea	11 and 21	10 (8 f/2 m)	46,4 (22 to 56)	11
Kan et al., 2009 [37]	CS	12 to 48	Loma Linda, CA	13 to 23	20 (14 f/6 m)	52,3 (18 to 71)	20
Kan et al., 2007 [38]	CS	12	Loma Linda, CA	13 to 23	23 (undefined)	39,5 (25 to 63)	23
Nemcovsky et al., 2000 [26]	CS	6 to 8	Tel Aviv, Israel	15 to 25	24 (undefined)	45,5 (29 to 65)	26
Tsuda et al., 2011 [39]	CS	12	Loma Linda, CA	14 to 24	10 (6 f/4 m)	48 (35 to 70)	10
Chung et al., 2011 [40]	CS	12	Loma Linda, CA	15 to 25 and 35 and 45	10 (4 f/6 m)	52,1 (22,7 to 67,1)	10
Covani et al., 2007 [20]	CS	12	Lucca, Italy	15 to 25 and 35 to 45	10 (5 f/5 m)	(42 to 55)	10
Jyothi et al., 2013 [21]	CS	12	Karnataka, India	15 to 25 and 35 to 45	10 (5 f/5 m)	25,3	10
Nemcovsky et al., 1999 [25]	CS	6 to 9	Tel Aviv, Israel	15 to 25	29 (undefined)	44,5	33

**Table 4:** Characteristics of the clinical procedure of included studies. N: no; Y: yes; SCTG: subepithelial connective tissue graft; IIPP: immediate implant placement and provisionalization; IIP: immediate implant placement; RPF: rotated palatal flap; TG: test group; CG: control group.

Authors, year	Buccal flap	Membrane	Xenograft	Allograft	Autogenous	Provisionalization on implant crown	Groups (sample)	Surgical technique soft tissue augmentation
Migliorati et al., 2015 [17]	N	N	Y	N	N	Y	TG (24), CG (24)	IIPP with SCTG (TG) and IIPP without SCTG (CG)
Bianchi and Sanfilippo, 2004 [6]	N	N	N	N	N	N	TG1 (32), TG2 (42),TG3 (22), and CG (22)	IIP with SCTG (3 TGs) and IIPP without SCTG (CG)
Yoshino et al., 2014 [18]	N	N	Y	N	N	Y	TG (10), CG (10)	IIPP with SCTG (TG) and IIPP without SCTG (CG)
Rungcharassaeng et al., 2012 [34]	N	N	Y	Y	N	N	TG (31), CG (24)	IPP with SCTG (TG) and without SCTG (CG)
Cornelini et al., 2008 [35]	Y/N	Y/N	N	N	N	Y	TG (17), CG (17)	IIPP with SCTG (TG) and IIPP without SCTG (CG)
Lee et al., 2012 [36]	Y	Y	Y	N	N	Y	TG (10)	IIPP with SCTG (TG)
Kan et al., 2009 [37]	N	N	Y	N	N	Y	TG (20)	IIPP with SCTG (TG)
Kan et al., 2007 [38]	Y/N	Y	Y	N	Y	Y	TG (11), CG (12)	IIPP with SCTG (TG) and IIPP without SCTG (CG)
Nemcovsky et al., 2000 [26]	Y	N	Y	N	N	N	TG (24)	IIP with RPF (TG)
Tsuda et al., 2011 [39]	N	Y	Y	N	N	Y	TG (10)	IIPP with SCTG (TG)
Chung et al., 2011 [40]	N	N	Y	N	N	Y	TG (10)	IIPP with SCTG (TG)
Covani et al., 2007 [20]	N	N	N	N	N	N	TG (10)	IIP with SCTG (TG)
Jyothi et al., 2013 [21]	Y	N	N	N	N	N	TG (10)	IIP with SCTG (TG)
Nemcovsky et al., 1999 [25]	Y	Y/N	Y	N	N	N	TG (14 in 15 sites), CG (15 in 18 sites)	IIP with RPF used membrane (TG) and IIP with RPF used no membrane (CG)

**Table 5:** Patient profile: inclusion and exclusion criteria.

Inclusion criteria	Exclusion criteria
Age ≥ 18 years	Systematic diseases that could alter tissue integration of dental implants (severe systematic problems)
Good oral hygiene	Pregnancy
Adequate native bone to achieve implant primary stability with sufficient bone volume with minimum dimensions of 3.3 × 12,0 mm or 3,25 × 15,0 mm or 3,5 × 13,0 mm	Alcohol or drug dependency
Presence of adequate gingival architecture with the surrounding dentition	Head and neck radiation treatment
Appropriate gingiva-to-underlying bone dimension facially (≥2 mm) and interproximally (4 to 6 mm)	Bruxism and/or parafunction
Adjacent teeth or implants without need for prosthetic restorations	A lack of stable posterior occlusion
Stable occlusion	Perforation and/or loss of the labial bony plaque after tooth removal and/or implant osteotomy
Adequate vertical dimension of the existing metal-ceramic prosthetic restorations	
Indications for periodontal treatment before the implant surgery	
Absence of periodontal disease	
Being without active infection	
Tobacco abuse	
No smoking	

**Table 6:** Survival rate of implant and survival rate of suitable aesthetic results. Total exposure time corresponds to the sum of exposure time of implants that survived the follow-up time, exposure time to the failure of implants lost during the observation time, and exposure time up to the end of follow-up time for the implants that did not complete the observation period due to any reason.

Study	Year	Total implants evaluated	Mean follow-up (years)	Number of failures of the implants	Total exposure time	Estimated survival rate of the implants (per 100 years)	Estimated rate of suitable aesthetic results (per 5 years)
Migliorati et al. [17]	2015	47	2	0	96	100%	65%
Bianchi and Sanfilippo [6]	2004	116	12	0	1392	100%	N/A
Yoshino et al. [18]	2014	20	1	0	20	100%	100%
Rungcharassaeng et al. [34]	2012	23	2,5	0	60	100%	N/A
Cornelini et al. [35]	2008	34	1	0	34	100%	32%
Lee et al. [36]	2012	11	2	0	20	100%	70%
Kan et al. [38]	2007	20	2,5	0	50	100%	84%
Kan et al. [37]	2009	23	1	0	23	100%	35%
Nemcovsky et al. [26]	2000	26	0,7	0	17	100%	N/A
Tsuda et al. [39]	2011	10	1	1	10	90%	30%
Chung et al. [40]	2011	10	1	1	10	90%	60%
Covani et al. [20]	2007	10	1	0	10	100%	N/A
Jyothi et al. [21]	2013	10	1	0	10	100%	N/A
Nemcovsky et al. [25]	1999	33	0,8	0	23	100%	N/A

Total	Fixed effects					6,018 (5,919–6,117)	1,261 (1,111–1,410)
	Random effects					6,526 (6,125–6,927)	1,292 (1,029–1,555)
	df					13	7
	*p* value					0,001	0,001
	*I* ^2^					93,21%	64,32%
